# Successful Management of Discordant Twin Growth in End-Stage Renal Disease: A Rare Case Report

**DOI:** 10.7759/cureus.112678

**Published:** 2026-07-14

**Authors:** Maha Wazir, Farhan Ullah, Salem Almaani, Udayan Bhatt

**Affiliations:** 1 Nephrology, The Ohio State University Wexner Medical Center, Columbus, USA; 2 Nephrology, Ochsner Medical Center, New Orleans, USA; 3 Nephrology, The Ohio State University College of Medicine, Ohio, USA

**Keywords:** esrd on hd, growth discordance, intensive hemodialysis, multiple gestation, preeclampsia, pregnancy in dialysis, renal failure, twin pregnancy, twin pregnancy in esrd

## Abstract

This case report describes the successful management of a dichorionic-diamniotic twin pregnancy in a 35-year-old woman with end-stage renal disease (ESRD) on hemodialysis. The patient had established kidney failure secondary to presumed hypertensive nephrosclerosis, complicated by a previous pregnancy with preeclampsia, and was maintained on home hemodialysis (five hours per session on alternate days) since 2022. Upon confirmation of twin pregnancy, her dialysis regimen was immediately intensified from alternate-day sessions to daily treatments totaling 35 hours/week, with parameters including five-hour duration, blood flow rate of 250 mL/min, and ultrafiltration of 1.2-1.5 kg/session. During the second trimester, maternal weight was carefully monitored alongside fetal growth, with an expected increase of 0.5-1 kg weekly. At 27 weeks’ gestation, significant fetal growth discordance prompted further dialysis intensification to six times weekly with increased blood and dialysate flow rates. The pregnancy was complicated by intrahepatic cholestasis and therapy-resistant hypertension. At 31 weeks, the patient developed acute pulmonary edema with severe preeclampsia features despite optimal volume management, requiring emergent cesarean delivery of two live infants. This case demonstrates that intensive dialysis protocols are essential for achieving favorable outcomes in twin pregnancies complicated by ESRD. Success depends on three critical components: early recognition of maternal and fetal complications, intensive fetal surveillance throughout pregnancy, and precise control of dialysis parameters with frequent adjustments based on clinical status and fetal development.

## Introduction

The journey of pregnancy for women with end-stage renal disease (ESRD) has evolved significantly since the first documented cases in the 1970s [1]. However, such pregnancies remain rare, with multiple pregnancies carrying substantial risks even for healthy individuals. This fact emphasizes how crucial it is to have family planning conversations with dialysis patients who are of childbearing age so that they possess the knowledge to make informed decisions [2].

The impact of renal disease on fertility is substantial. Kidney transplant recipients generally experience better fertility and pregnancy outcomes compared to women on dialysis, though both groups face significant challenges. Specifically, transplant recipients demonstrate approximately 1/10th of the general population's fertility rate, while women on dialysis show even lower rates at about 1/100th [3,4]. Among women on dialysis therapy, conception rates range from 1% to 7.9% during childbearing years, though recent advances in hemodialysis techniques have led to encouraging improvements in pregnancy rates [5]. ESRD leads to reduced estradiol levels and decreased prolactin clearance, which interferes with both gonadotropin-releasing hormone pulsatility and the crucial midcycle surge of luteinizing hormone necessary for ovulation [6]. Importantly, when conception does occur in a woman already dependent on dialysis, the physiologic burden of pregnancy is superimposed on a state of minimal or absent residual renal function. This burden is magnified substantially in multiple gestations, which impose additional demands on maternal cardiac output, plasma volume expansion, and solute clearance beyond what is required in a singleton pregnancy. As a result, approximately 70% of women on hemodialysis experience oligo- or amenorrhea [7]. Women with ESRD on dialysis face significantly higher risks during pregnancy, including preterm delivery, cesarean section, blood transfusion requirements, and severe maternal complications. A comprehensive multidisciplinary approach to family planning has proven effective, with 90% of women reporting that the integrated support enabled them to make informed pregnancy decisions [8]. Twin pregnancies in dialysis-dependent women remain exceedingly rare and are addressed by only a handful of published reports, which sets up the rationale for the case report.

## Case presentation

Our patient is a 35-year-old gravida 2 para 1 with a past medical history of hypertension, preeclampsia eight years previously, an atrophic right kidney (diagnosed 10 years previously), a prior cesarean section, and a distant appendectomy. She had a family history of hypertension in her father but no renal disease.

First pregnancy and initial chronic kidney disease diagnosis

She was diagnosed with chronic kidney disease (CKD) stage 3b before being seen in our institution 10 years previously. She was noted to be 27 weeks pregnant at that time with preeclampsia. Laboratory findings across all four clinical encounters are summarized in Table 1. Baseline serum creatinine was 1.97 mg/dL (estimated glomerular filtration rate: 31 mL/min/1.73 m²), with a urine protein-to-creatinine ratio (UPCR) of 2 g/g. Renal duplex showed decreased right kidney size (6.14 cm) compared with the left kidney (11.6 cm) and minimal pelvocaliectasis of the left kidney without significant hydronephrosis. CKD was presumed to be due to hypertension. Her systolic blood pressure at the time was 140-150 mmHg, and she was maintained on labetalol and amlodipine. Initial serologic testing was negative, with no monoclonal proteins noted in the urine protein electrophoresis. A 24-hour urine protein was requested but not completed. Renal biopsy was recommended; however, the patient declined. She delivered a healthy child (weight: 1460 g and Apgar: 9/9) at 34 weeks and 1 day via lower segment cesarean section complicated by placental abruption. During this time, her creatinine increased to 2.5 mg/dL with a UPCR of 6.233 g/g. She was maintained on labetalol 300 mg BID and amlodipine 10 mg daily. She was then lost to follow-up.

**Table 1:** Laboratory findings across clinical time points ACOG, American College of Obstetricians and Gynecologists; AKI, acute kidney injury; ANA, antinuclear antibody; ANCA, antineutrophil cytoplasmic antibody; BUN, blood urea nitrogen; dsDNA, double-stranded deoxyribonucleic acid; EF, ejection fraction; ENA, extractable nuclear antigen; eGFR, estimated glomerular filtration rate; GBM, glomerular basement membrane; Hgb, hemoglobin; JO-1, histidyl-tRNA synthetase antibody; RNP, ribonucleoprotein; SCL-70, scleroderma antibody (topoisomerase I); SCr, serum creatinine; SM, Smith antigen; SSA/B, Sjögren syndrome antigen A/B; TP, time point; TSAT, transferrin saturation; UPCR, urine protein-to-creatinine ratio; UPEP, urine protein electrophoresis; —, not measured at this time point. Note: Reference ranges apply to non-pregnant adult females. Serum creatinine values at TP3 reflect the pre-admission baseline and peak during hospitalization. Reference ranges apply to non-pregnant adult females unless otherwise specified. Pregnancy ranges for TP4 are less directly applicable given the patient's minimal residual native glomerular filtration rate (end-stage renal disease); the physiologic mechanism (glomerular filtration rate augmentation) driving pregnancy reference ranges does not apply in the same way to a dialysis-dependent patient. [9]

Test	TP1 (first pregnancy visit)	TP2 (at delivery)	TP3 (AKI hospitalization)	TP4 (second pregnancy – inpatient stay)	Non-pregnant reference range	Pregnancy reference range
Renal function						
SCr	1.97	2.5	2.3-3.0 (baseline)/15.18 (peak)	—	0.5-1.1 (mg/dL)	T1: 0.4-0.7 · T2: 0.4-0.8 · T3: 0.4-0.9 (mg/dL)
eGFR	31	—	—	—	≥60 (mL/min/1.73 m²)	No validated pregnancy-specific eGFR reference range; standard equations not validated in pregnancy
BUN	—	—	—	3-6	7-20 (mg/dL)	T1: 7-12 · T2: 3-13 · T3: 3-11 (mg/dL)
Urine studies						
UPCR	2	6.233	4.1	—	<0.2 (g/g)	Upper limit for pathologic proteinuria in pregnancy ~0.3 g/g; mild GFR-driven increases in normal pregnancy not well quantified by UPCR specifically
24-hour urine protein	Not completed	—	1.17	—	<0.15 (g/day)	<0.3 g/day (ACOG threshold for pathologic proteinuria in pregnancy)
Hematologic and iron studies						
Hgb	—	—	7.7	9.2-9.9	12.0-16.0 (g/dL)	T1: 11.6-13.9 · T2: 9.7-14.8 · T3: 9.5-15.0 (g/dL)
Serum iron	—	—	29	—	60-170 (mcg/dL)	No distinct validated pregnancy range; interpret with non-pregnant range
TSAT	—	—	10	—	20-50 (%)	No distinct validated pregnancy range
Serum ferritin	—	—	28	—	12-150 (ng/mL)	Ferritin <30 ng/mL supports iron deficiency in pregnancy (lower clinical threshold), though assay reference range unchanged
Metabolic						
Serum phosphorus	—	—	—	2.6-5.3	2.5-4.5 (mg/dL)	No clinically distinct pregnancy range
Serum potassium	—	—	—	3.9-4.6	3.5-5.0 (mEq/L)	No clinically distinct pregnancy range
Cardiac						
EF	—	—	45-50	—	55-70 (%)	No distinct validated pregnancy reference range for EF itself, though cardiac output and stroke volume increase
Serologic and immunologic						
ANA	Negative	—	Negative	—	Negative/unremarkable	No pregnancy-specific reference range -- interpreted identically in pregnancy
Anti-dsDNA	Negative	—	Negative	—	Negative/unremarkable	
Serum complements	Unremarkable	—	Unremarkable	—	Negative/unremarkable	
ANCA	Negative	—	Negative	—	Negative/unremarkable	
Anti-GBM	Negative	—	Negative	—	Negative/unremarkable	
ENA screen (SSA/B, SM, RNP, SCL-70, JO-1)	Negative	—	Negative	—	Negative/unremarkable	
UPEP	Negative	—	—	—	Negative/unremarkable	
Chronic hepatitis serologies	Negative	—	Negative	—	Negative/unremarkable	

Interval acute kidney injury hospitalization and transition to hemodialysis

She was admitted four years afterward at an outside hospital for oliguric acute kidney injury on CKD III (baseline creatinine had been 2.3-3 mg/dL the year before), complicated by small-to-moderate pericardial effusion (ejection fraction: 45%-50%) with peak creatinine of 15.18 mg/dL. Urine sediment showed dysmorphic RBC casts and mixed cellular casts supporting a possible glomerulonephritis diagnosis. Her UPCR was 4.1 g/g, but the 24-hour urine protein was 1.17 g. Iron-deficiency anemia was also evident with a transferrin saturation of 10%, iron of 29 mcg/dL, ferritin of 28 ng/mL, and a hemoglobin (Hgb) of 7.7 g/dL. She was also noted to have secondary hyperparathyroidism, vitamin D deficiency, and presumptive anemia of CKD. Antinuclear antibody, anti-double-stranded deoxyribonucleic acid, serum complements, antineutrophil cytoplasmic antibody, anti-glomerular basement membrane, extractable nuclear antigen screen (SSA/B, SM, RNP, SCL 70, JO1), and chronic hepatitis serologies were again unremarkable. She underwent renal biopsy in January 2019, which showed small fragments of fibrotic renal parenchyma suggestive of advanced-stage chronic renal injury. Unfortunately, the biopsy specimen was limited, and it was not possible to determine the underlying etiology. A broad differential was possible with a genetic form of renal disease, primary glomerular, tubulointerstitial disease, and, less likely, a primary vascular disease could have been possible. Chronic hemodialysis three times a week was initiated. She was evaluated for a kidney transplant later that year but was taken off the list due to an incomplete work-up. After three years of in-center dialysis, she was switched to home hemodialysis. Her home hemodialysis schedule was set at dialysis every other day, and each session ultrafiltration was about 1 to 2.2 L (roughly 2 to 5 pounds) of excess fluid from her body.

Second pregnancy: dialysis intensification

She had her second pregnancy two years later with a dichorionic-diamniotic twin pregnancy. At about 16 weeks, she was again seen in the home hemodialysis clinic. After the twin pregnancy was noted, her dialysis prescription was increased to seven days/week, five hours/day, a blood flow rate (BFR) of 250 cc/min, heparin administration via pump at 1 mL/h, and an ultrafiltration rate (UFR) of 4.5 cc/kg/h. Because of a drop in Hgb, her Mircera was switched to erythropoietin (20,000 units twice weekly). She was noted to be hypokalemic; thus, supplementation was started. She was maintained on prenatal vitamins and low-dose aspirin.

Delivery and postpartum course

Subsequently, she was diagnosed with intrahepatic cholestasis of pregnancy and maintained on ursodiol and diphenhydramine as needed. She followed with maternal-fetal medicine with good fetal growth until 27 weeks and 3 days gestational age, when fetus B was noted to have severe fetal growth restriction and absent end-diastolic flow of the umbilical artery, while fetus A was noted to have mild polyhydramnios. She was admitted for inpatient management for ongoing fetal surveillance with planned delivery at 33-34 weeks. Neonatology and nephrology were consulted. Following admission, fetal surveillance consisted of daily biophysical profiles and umbilical artery Doppler studies for fetus B, with serial growth ultrasounds performed every week to assess for progression of discordant growth and to guide timing of delivery. Daily dialysis except Sunday was planned with a five-hour duration, 4 mEq/L K bath, a BFR of 350 cc/min, a dialysate flow rate of 700-800 cc/min, and an ultrafiltration volume based on daily weight (difference between post-hemodialysis and pre-hemodialysis weight the following day, utilizing a target estimated dry weight of 53 kg). Heparin 2000 units was given mid-treatment. Potassium chloride 20 mEq PO BID, low-dose aspirin, prenatal vitamins, and renal vitamins were continued. Blood urea nitrogen values ranged from 3 to 6 mg/dL, phosphorus levels varied between 2.6 and 5.3 mg/dL without phosphate binders, potassium varied between 3.9 and 4.6mEq/L, and Hgb was between 9.2 and 9.9 g/dL. She was given a total of 1 g ferric gluconate for relative iron deficiency. Erythropoietin was advanced to 12,000 -14,000 units thrice weekly. Blood pressure was controlled without medications. Labs were monitored every Monday, Wednesday, and Friday. At 29 weeks and 5 days, her systolic blood pressure continued to rise to 130-150 mmHg. Labetalol 200 mg TID was started and titrated upward. Nifedipine was added, as her blood pressure increased up to 170 mmHg. She also had increased swelling with occasional shortness of breath, especially after her day off dialysis. Therefore, her UFR was increased from 1.8 to 5.7 cc/kg/h. At 31 weeks and 2 days, she developed orthopnea with increased swelling and pulmonary edema on chest X-ray. There was concern for worsening preeclampsia. She was given magnesium sulfate as a 2 g bolus followed by a maintenance drip of 1 g/h. The decision was made to deliver the infants. She delivered operatively to a live male (infant A) in breech presentation with a birthweight of 2350 g and a live female (infant B) in breech presentation with a birthweight of 1950 g. Both infants were admitted to the neonatal intensive care unit given their prematurity. No respiratory support was needed, and both underwent standard preterm screening, including cranial ultrasound and hearing assessment, which were unremarkable. Both infants were discharged home on day 7 of life in stable condition. Mother was returned to thrice weekly hemodialysis postpartum. Nifedipine 60 mg BID and labetalol 600 mg TID were continued on discharge, with blood pressure stabilizing to goal <130 by approximately one week postpartum, at which point antihypertensive therapy was tapered to nifedipine 30 mg BID.

## Discussion

The management of twin pregnancies in patients receiving maintenance hemodialysis presents one of the most complex challenges in maternal healthcare. Although pregnancy outcomes in dialysis-dependent patients have shown promising improvements with success rates reaching 70% [4,10], twin pregnancies introduce additional layers of complexity. The better pregnancy outcomes have been largely attributed to intensive hemodialysis regimens of approximately 43 ± 6 hours per week [5].

These pregnancies not only occur infrequently but also carry substantially elevated risks for both maternal and fetal complications compared with single pregnancies, as both conditions independently contribute to adverse pregnancy outcomes [11]. The primary complications include premature delivery and the potential for growth restriction affecting one or both fetuses [11,12]. In order to improve outcomes for both mothers and newborns, this combination of risk factors necessitates extraordinary monitoring and expert medical intervention. Strategic protocol changes are necessary. In our case, upon discovering the twin gestation, we implemented an intensive dialysis schedule of 35 hours weekly across seven days. Studies have demonstrated that lowering blood urea nitrogen levels below 35 mg/dL or giving patients with no residual renal function at least 36 hours/week of dialysis is associated with higher live birth rates and longer gestational periods. Home-based dialysis often proves most practical for implementing such intensive schedules. The twin pregnancy scenario also necessitates careful adjustment of dialysate composition to accommodate both increased clearance from frequent dialysis and fetal developmental needs [13].

Monitoring and regular adjustments are necessary to manage anemia in twin pregnancies receiving dialysis. Our experience demonstrated the need to switch from Mircera to erythropoietin for more precise dose control. Current guidelines recommend increasing erythropoietin-stimulating agents to two to three times the pre-pregnancy dose to maintain hemoglobin levels between 10 and 11 mg/dL [13]. In our case, supplemental ferric gluconate was necessary to maintain adequate iron stores. Also, nutritional management is equally crucial. Pregnant women on dialysis require increased protein intake of 1.5-1.8 mg/kg/day, necessitating close dietary supervision. Proper fetal bone development demands careful monitoring of maternal calcium and phosphorus levels, often requiring adjustments to calcium dialysate concentration and phosphorus supplementation. While calcium-based binders and vitamin D analogs are considered safe during pregnancy when maternal calcium levels remain normal, sevelamer should be avoided due to its association with irregular fetal bone ossification in animal studies [13].

A critical turning point in our case occurred at 27 weeks of gestation, when we detected discordant fetal growth. One twin exhibited severe growth restriction with absent end-diastolic flow, while the other developed mild polyhydramnios. This complex presentation necessitated hospital admission for intensive monitoring, highlighting how maternal renal disease can differently affect twin development.

Similar challenges have been documented in other cases. One report describes a twin pregnancy in a woman with stage 4 CKD who required hemodialysis initiation at 24 weeks due to polyhydramnios and growth restriction complications. Her case resulted in delivery at 28 weeks following preeclampsia development [14]. In another remarkable case, a successful triplet pregnancy was achieved in a dialysis patient, with a gradual increase in dialysis duration to 18.75 hours weekly, resulting in live births at 34 weeks [15]. Perhaps most notably, one case documents a woman who managed four pregnancies while on dialysis, including a twin pregnancy, with her final pregnancy occurring during her 14th year of hemodialysis treatment [12].

Women on dialysis face a significantly elevated risk of preeclampsia, occurring in 5%-20% of pregnancies [13]. The clinical challenge lies in differentiating between preeclampsia and volume overload in ESRD patients. When patients experience rapid worsening of hypertension alongside other preeclampsia indicators, early delivery should be considered [4]. This diagnostic challenge was evident in our case, where progressive hypertension ultimately led to pulmonary edema, compelling us to proceed with delivery.

In pregnancies complicated by ESRD, fetal growth restriction remains a significant clinical challenge [11]. While intensive dialysis protocols have shown promise in mitigating growth restriction risks, the underlying mechanisms continue to be an area of active research [5]. The unique presentation in our case -- where one twin experienced severe growth restriction while the other maintained normal growth -- adds to the limited literature on growth discordance in twin pregnancies with ESRD. This distinct pattern emphasizes how maternal renal disease can differently impact fetal development in multiple gestations.

In managing pregnant ESRD patients, volume control presents a significant clinical challenge, particularly in twin pregnancies. While singleton pregnancies have established guidelines recommending weekly dry weight increases of 0.5 kg during the second and third trimesters, twin pregnancies lack specific protocols. Current literature emphasizes the need for more rigorous volume status monitoring in twin gestations, with ultrafiltration goals requiring frequent adjustments based on both clinical assessment and fetal monitoring parameters [16,17].

Recent studies have shown encouraging improvements in live birth rates among women on hemodialysis, reaching 40%-50%. However, neonatal mortality remains a significant concern, ranging from 8% to 13% [18,19]. Successful management of these pregnancies requires a comprehensive approach. Evidence-based guidelines for renal replacement therapy during pregnancy emphasize multiple critical factors: pre-conception creatinine assessment, intensified dialysis schedule (five to seven sessions weekly), maintaining urea levels between 30 and 50 mg/dL, careful management of erythropoietin to achieve hemoglobin levels of 10-11 g/dL, rigorous control of electrolytes and blood pressure, proactive prevention of urinary tract infections, and regular fetal monitoring [19]. Essential to success is the coordination of care through a specialized referral center with a multidisciplinary team comprising obstetricians, nephrologists, and nutritionists. Despite these measures, research has identified three predominant complications: worsening maternal hypertension, polyhydramnios, and preterm labor [20].

## Conclusions

This case demonstrates that successful outcomes in twin pregnancies on maintenance hemodialysis are achievable through vigilant clinical management. Given that one twin in our case had significant growth limitation, while the other twin continued to develop normally, the particular difficulty of discordant fetal growth underscores the intricate relationship between maternal renal illness and multiple gestations. The successful management relied on three key elements: early recognition of complications, frequent fetal surveillance, and precise control of dialysis parameters. This experience contributes valuable insights to the limited literature on multiple gestations in dialysis-dependent patients, particularly regarding the management of discordant growth. As more such cases are documented, we anticipate the development of more comprehensive guidelines specifically addressing the unique challenges of twin pregnancies in this high-risk population.
